# Confirmation of Oryctes rhinoceros nudivirus infections in G-haplotype coconut rhinoceros beetles (*Oryctes rhinoceros*) from Palauan PCR-positive populations

**DOI:** 10.1038/s41598-021-97426-w

**Published:** 2021-09-20

**Authors:** Shunsuke Tanaka, Robert L. Harrison, Hiroshi Arai, Yukie Katayama, Tetsuya Mizutani, Maki N. Inoue, Joel Miles, Sean D. G. Marshall, Christopher Kitalong, Madoka Nakai

**Affiliations:** 1 Tokyo University of Agriculture and Technology, Saiwai-cho, Fuchu-shi, Tokyo, 183-8509 Japan; 2 Invasive Insect Biocontrol and Behavior Laboratory, Beltsville Agricultural Research Center, USDA Agricultural Research Service, 10300 Baltimore Avenue, Beltsville, MD 20705 USA; 3 Palau National Invasive Species Coordinator, Retired, Koror, Palau; 4 AgResearch Limited (Lincoln), Research Centre, Private Bag 4749, Christchurch, Lincoln, 8140 New Zealand; 5 Palau Community College-Cooperative Research Extension, Koror, Palau; 6 Pacific Academic Institute for Research, Koror, Palau

**Keywords:** Ecosystem ecology, Virus-host interactions

## Abstract

Coconut rhinoceros beetle (CRB), *Oryctes rhinoceros*, is a pest of palm trees in the Pacific. Recently, a remarkable degree of palm damage reported in Guam, Hawaii, Papua New Guinea and Solomon Islands has been associated with a particular haplotype (clade I), known as “CRB-G”. In the Palau Archipelago, both CRB-G and another haplotype (clade IV) belonging to the CRB-S cluster coexist in the field. In this study, more than 75% of pheromone trap-captured adults of both haplotypes were Oryctes rhinoceros nudivirus (OrNV)-positive by PCR. No significant difference in OrNV prevalence between the haplotypes was detected. In PCR-positive CRB-G tissue specimens from Palau, viral particles were observed by electron microscopy. Hemocoel injection of CRB larvae with crude virus homogenates from these tissues resulted in viral infection and mortality. OrNV isolated from Palauan-sourced CRB was designated as OrNV-Palau1. Both OrNV-Palau1 and OrNV-X2B, a CRB biological control isolate released in the Pacific, were propagated using the FRI-AnCu-35 cell line for production of inoculum. However, the OrNV-Palau1 isolate exhibited lower viral production levels and longer larval survival times compared to OrNV-X2B in *O. rhinoceros* larvae. Full genome sequences of the OrNV-Palau1 and -X2B isolates were determined and found to be closely related to each other. Altogether these results suggest CRB adults in Palau are infected with a less virulent virus, which may affect the nature and extent of OrNV-induced pathology in Palauan populations of CRB.

## Introduction

Coconut palms, often referred to as the “tree of life”^1^ in the Pacific, provide numerous benefits to human society. In the Pacific, the coconut rhinoceros beetle (CRB), *Oryctes rhinoceros* (Linnaeus, 1758) (Coleoptera: Scarabaeidae: Dynastinae) has caused serious damage to palms, including coconut and oil palms. Adults of CRB burrow into the crown of a palm to mainly feed on the sap. As the meristem of palm is in the crown, burrowing activity commonly damages developing palm fronds, which then generally display a characteristic “V-shaped” notching pattern once unfurled. This feeding action leads to reductions in both coconut palm growth and nut production due to a reduction of photosynthesis efficiency, and can cause death if the meristem itself is damaged^2^. Furthermore, adult female beetles lay eggs in dead palms, and the hatched larvae feed on the decomposing palm materials^3^. Thus, CRB uses the coconut palms as a resource during all of its developmental stages, though they can use alternative food sources as well.

To manage outbreaks of CRB, various control campaigns were conducted^4,5^. Because control with chemical insecticides was ineffective and unsuitable due to labor costs and negative effects on both humans and the environment, control of CRB has relied on natural enemies, particularly Oryctes rhinoceros nudivirus (OrNV)^6^. Control with OrNV involves releasing adult beetles inoculated with OrNV into CRB infested areas^7^. OrNV then is transmitted among individuals in an infesting population by feeding on food contaminated with OrNV-containing feces of infected beetles, and also during mating with infected insects^8^. The introduction of OrNV-infected beetles into palm-growing sites in the Pacific beginning in the late 1960s was a successful case of classical biological control and successfully reduced palm damage^2^.

However, a CRB population with tolerance to OrNV recently appeared in Guam and spread throughout the island^9,10^. Control attempts with commonly released OrNV biocontrol isolates were unsuccessful. Marshall et al. (2017) found that the Guam population had a distinguishing nucleotide substitution in the mitochondrial COI gene and designated this new haplotype as CRB-G (clade I), to distinguish it from other populations which were designated CRB-S (clades II, III, and IV)^9^. The CRB-G haplotype has since been identified in other Pacific locations such as Hawaii, Papua New Guinea, and Solomon Islands^10^.

In the Palau archipelago of Micronesia, the population of CRB is a distinctive mixture of both CRB-G and CRB-S adults analyzed by PCR^9^. This implies that the new CRB-G haplotype can invade other countries and regions occupied by other CRB haplotypes. A high prevalence of OrNV has been detected from pheromone trap-captured adults analysed by PCR. However, other nudiviruses have been reported to integrate copies of viral genomes into the chromosomes of their hosts, which may also yield a positive result for presence of virus by PCR without actually indicating a virus infection^11^. Nudivirus infection sometimes causes swelling of the gut in adults, but this symptom is also not a reliably accurate marker of OrNV infection. Hence, the pathological activity and the OrNV genome organization of infected CRB from Palau remains to be determined^9^. Understanding the ecosystem of viruses and beetles in Palau may provide important insights into palm conservation with mixed-haplotype CRB populations in the Pacific regions and elsewhere in South and Southeast Asia.

In this study, the presence of OrNV in field-trapped CRB from Palau was determined by PCR and transmission electron microscopy. Virus was extracted from Palau CRB and evaluated for pathogenicity by bioassays with CRB larvae sourced in Japan. Replication and the full genome sequence of OrNV-Palau1 were compared to those of a commonly used biological control agent, the OrNV-X2B isolate.

## Results

### Haplotypes and virus detection in Palauan population

Adult CRBs were captured by aggregation pheromone traps in the Babeldaob and Koror islands. Their haplotypes and the presence of OrNV sequences were determined by PCR. According to *COI* gene sequences, 48 out of 80 adults were CRB-G and the rest were CRB-S (Table 1). Of these 80 adults, 62 were positive for OrNV (77.5%). Among the CRB-G adults, 38 out of 48 were positive (79.2%). Of the CRB-S adults, 24 out of 32 were positive (75.0%). There was no significant difference between the prevalence of OrNV in CRB-G and CRB-S (χ^2^ test, χ^2^ = 0.191, *p* = 0.662).

**Table 1:** Haplotype and detection of virus in *Oryctes rhinoceros* adults captured in the Palau Archipelago.

Location		No	G-type	Viral detection		S-type	Viral detection		OrNV + (%)
Latitude	Langitude			OrNV +	OrNV−		OrNV +	OrNV−	
Aimeliik 44.2248, 82.3112		8	2	2	0	6	6	0	100
Airai 45.1247, 81.6149		6	3	3	0	3	3	0	100
Echang 43.9509, 81.2162		11	7	4	3	4	1	3	45.5
Long 43.9339, 81.0006		11	6	2	4	5	2	3	36.4
Melekeok 45.9918, 82.8202		3	2	2	0	1	1	0	100
Ngaraard 46.0755, 84.3157		8	6	6	0	2	2	0	100
Ngerermlengui 44.9704, 83.0154		7	5	5	0	2	2	0	100
Ngetmeduch 44.4588, 81.3443		9	6	5	1	3	3	0	88.9
Tiull 44.2068, 81.1267		17	11	9	2	6	4	2	76.5
Total		80	48	39	9	32	24	8	77.5

(1) Number of adults examined.

(2) OrNV + : Number of samples showed OrNV-positive detection by PCR, OrNV−: Number of samples showed OrNV-negative detection by PCR.

**Figure 1:**
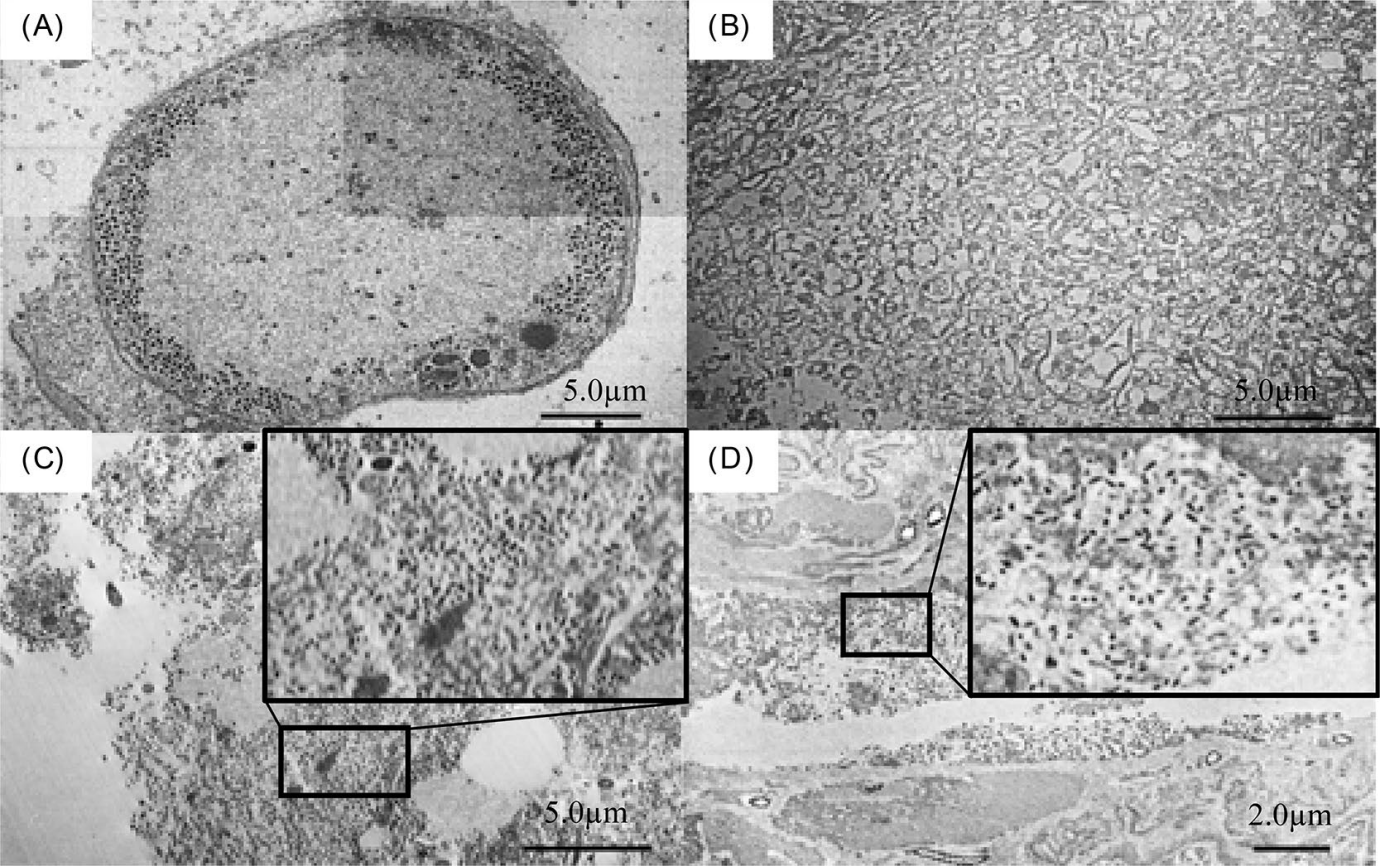
Electron micrographs of tissues of Palauan CRBs. (**A**) Japanese CRB adult was injected with the OrNV-X2B isolate as a positive control; fat body, (**B**) Japanese healthy untreated CRB adult; fat body, (**C**) Palauan field-collected CRB adult; fat body, (**D**) Palauan field-collected CRB adult; midgut. Magnifications were as follows. (**A**) ×4000, (**B**) ×2000, (**C**) ×2000, (**D**) ×3000. Black insets in (**C**,**D**) are higher-magnification images of virus particles (white arrows).

Virus particles were detected in the midgut and fat body from field-captured Palauan adults by transmission electron microscopy (TEM). Rod shaped particles were observed in two Palauan individuals as well as a Japanese adult injected with the OrNV-X2B isolate to provide a positive control (Fig. 1). The shape and size of these viral particles were consistent with previous descriptions of OrNV^12^. No OrNV-like particles were observed in a mock-infected negative control Japanese adult.

### Infectivity of OrNV Palau to Japanese CRB larvae

**Figure 2:**
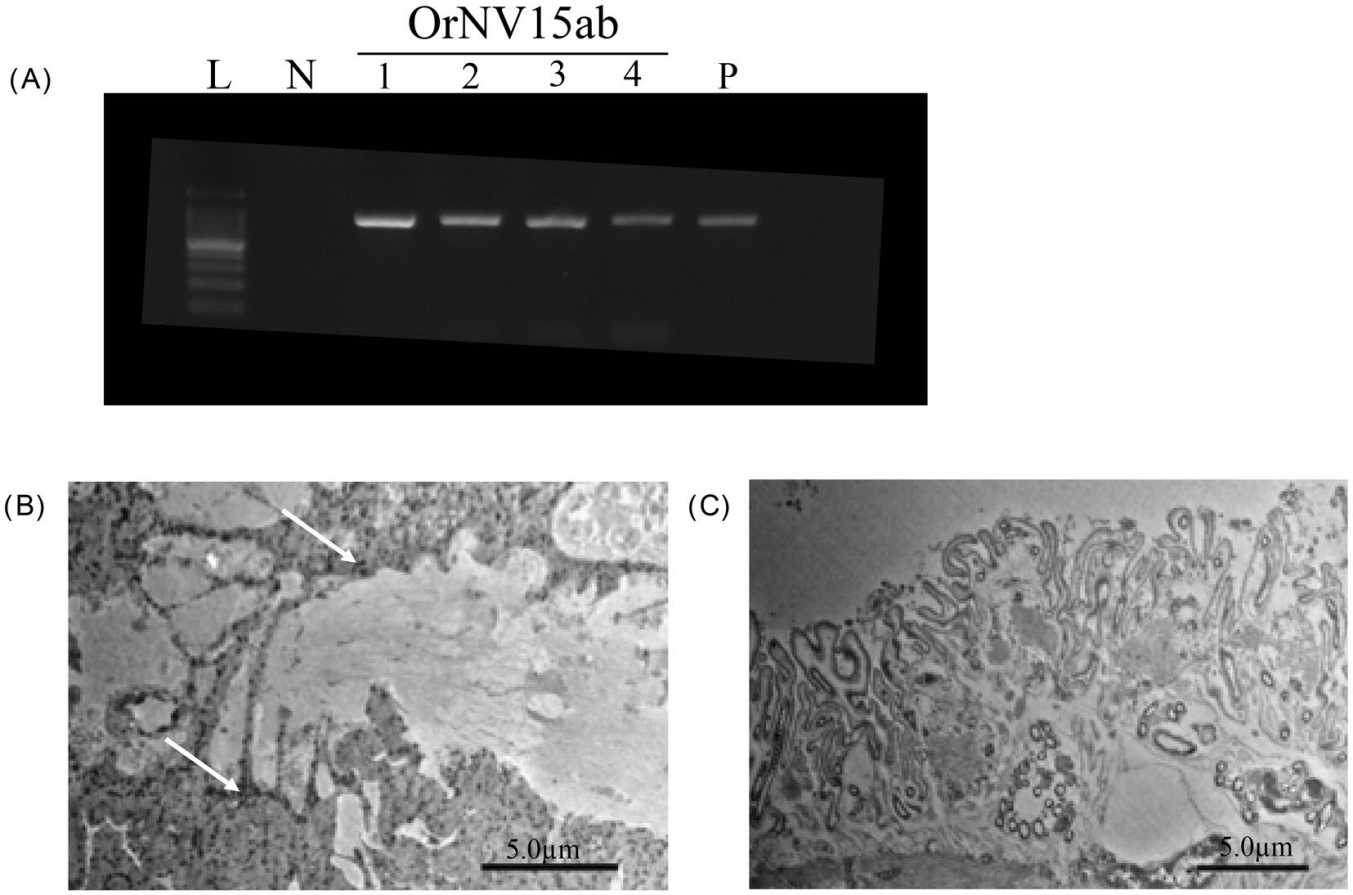
Expression of viral gene and virus particles in Japanese larvae inoculated with Palauan isolate. **(A**) Detection of OrNV RNA expression from midgut tissue of Japanese *O. rhinoceros* larvae inoculated with virus solution. L: 100 bp DNA Ladder (TaKaRa, Japan), N: Negative control (water) amplified by primers of OrNV15ab 1–4: individuals injected with virus solution, P: Positive control for PCR (DNA extracted from *O. rhinoceros-*infected adult). (**B**) Electron micrographs of tissues from Japanese *O. rhinoceros* larvae injected with virus solution, and (**C**) mock treatment (injected with PBS). Scale bars for micrographs shown with images. White arrows indicate virus particles.

Infectivity of a crude OrNV preparation from two infected Palauan (Melekeok) CRB-G adults was assessed following haemocoelic injection into healthy Japanese CRB larvae. Seven out of 8 larvae injected with crude virus extract died in 14 days post infection (dpi) with the characteristic OrNV-induced pathology described previously (Huger, 1966), including swollen midguts and prolapsed hindguts. Viral gene mRNA was detected from all injected samples by reverse transcription PCR (RT-PCR) (Fig. 2A). In addition, virus particles were observed in the midgut from an injected larva using TEM (Fig. 2B).

### Inoculum preparation using FRI-AnCu-35 cells

To determine the infectivity of OrNV to FRI-AnCu-35 (AnCu35) cells, we observed cells inoculated with the OrNV-X2B isolate every day for 9 dpi. A cytopathic effect (CPE) in the form of cell rounding was detected after 5 dpi (Fig. S1A), but was not detected in mock infected AnCu35 cells (Fig. S1B). Thus, infection of AnCu35 by OrNV was confirmed.

AnCu35 cells were also inoculated with the crude virus extract from Melekeok adults, and progeny virus from this infection, designated as isolate OrNV-Palau1, was used in downstream experiments.

To quantify the titer of the virus propagated in AnCu35 cells, the viral genome copies in 1 ng of total DNA extracted from cells inoculated with OrNV-Palau1 or -X2B were measured by quantitative PCR (qPCR). The average copy number of the OrNV-Palau1 and -X2B inoculants were 3.1 × 10^5^ and 3.3 × 10^5^ copies/ng total DNA, respectively.

### Time course of viral replication and killing speed of Palauan isolates in CRB larvae

**Figure 3:**
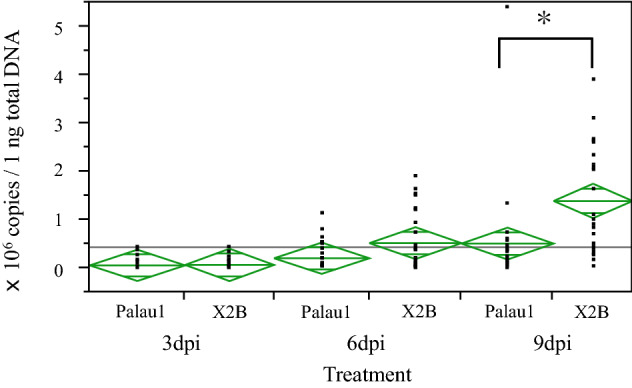
Viral copies in 1 ng total DNA extracted from Japanese larvae injected with OrNV Palau1 isolate and OrNV-X2B isolate, respectively. Center lines of green diamonds indicated average copy number, and a length between upper and lower point of diamond indicate the 95% confidence interval. Between two strains at each time points, the viral copies of OrNV-X2B were significantly higher than those of OrNV-Palau1 at 9 dpi (Steel–Dwass test, Z = 3.460, *p* = 0.0071: * indicated significant difference).

To evaluate viral replication in Japan-sourced second instar CRB larvae, viral copies in 1 ng of total DNA extracted from larvae hemocoelically injected with OrNV-Palau1 or OrNV-X2B were measured by qPCR. We obtained 300–3000 ng/µl DNA from inoculated larvae. At 3, 6, and 9 dpi, the average viral copies of OrNV-Palau1 were 6.0 × 10^4^, 2.1 × 10^5^, 5.1 × 10^5^ copies/ng total DNA, respectively (Fig. 3). The genome copy number significantly increased with time (Steel–Dwass test, 3 dpi vs. 6 dpi : Z = 3.47, *p* = 0.0017, 3 dpi vs. 9 dpi : Z = 5.12, *p* < 0.0001, 6 dpi vs. 9 dpi : Z = 2.77, *p* = 0.0155). The average viral copies of OrNV-X2B were 6.8 × 10^4^, 5.2 × 10^5^, 1.4 × 10^6^ copies/ng total DNA at 3, 6, and 9 dpi, respectively (Fig. 3). As with OrNV-Palau1, genome copy numbers for X2B significantly increased with time (Steel–Dwass test, 3 dpi vs. 6 dpi : Z = 4.12, *p* = 0.0001, 3 dpi vs. 9 dpi : Z = 5.58, *p* < 0.0001, 6 dpi vs. 9 dpi : Z = 3.26, *p* = 0.0032). The viral genome copy number of OrNV-X2B was significantly higher than that of OrNV-Palau1 at 9 dpi (Steel–Dwass test, Z = 3.460, *p* = 0.0071). At 3 and 6 dpi, there was no significant difference in copy numbers between the two isolates (Steel–Dwass test, 3 dpi : Z = 1.43, *p* = 0.1667, 6 dpi : Z = 2.37, *p* = 0.7108).

The killing speed of the Palauan isolate was examined by haemocoelic injection as described above. Median survival times of CRB larvae inoculated with OrNV-Palau1 and -X2B were 12 and 10 days, respectively. OrNV-Palau1 killed CRB larvae significantly more slowly than X2B (Wilcoxon test, *p* < 0.0001, chi-square = 50.0947). Larvae inoculated with PBS as a mock infection treatment did not begin to die until 35 dpi.

### Genome sequences

**Figure 4:**
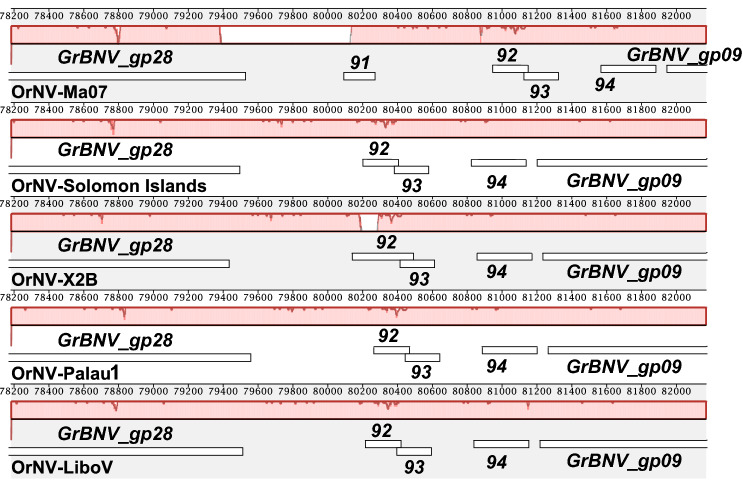
Mauve alignment of a region in OrNV genomes characterized by the deletion of a region containing OrNV-Ma07 ORF91. The numbering of ORFs in the OrNV-Ma07 genome is used to indicate ORFs that are conserved among isolates Ma07, Solomon Islands, X2B, Palau1, and LiboV. The gap in the block outline of the Locally Collinear Block (LCB) of Ma07 indicates the stretch of sequence in Ma07 which is not conserved in the alignment consensus sequence due to being missing from the other genomes.

**Figure 5:**
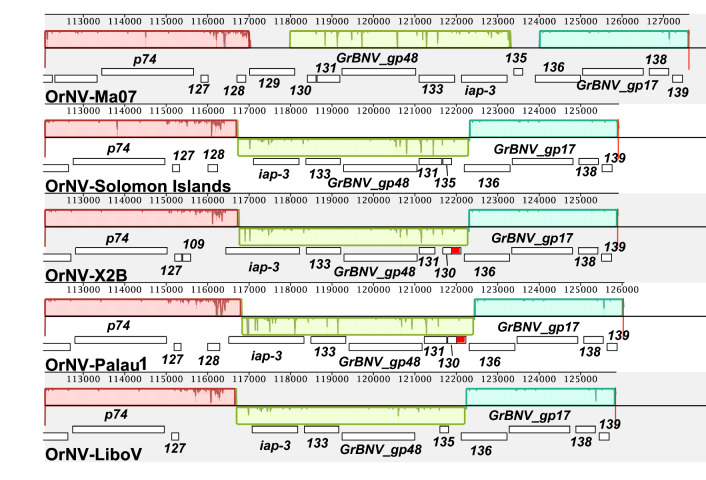
Mauve alignment of a region in OrNV genomes characterized by an inversion. Block outlines of the same color correspond to Locally Collinear Blocks (LCBs) which represent conserved segments of sequence among the isolates. The light green LCB represents the region in Ma07 which is inverted in the other isolates, which is indicated by this LCB occurring below the central tracking line in the Solomon Islands, X2B, Palau1, and LiboV sequences. The regions outside the LCBs lack significant sequence conservation with the alignment consensus and indicate sequences in Ma07 that are missing in the other isolates. Conserved ORFs are indicated by their specific names or by their numbering in the Ma07 genome. The red ORF was identified in the X2B and Palau1 genome sequences but either is not present (Ma07, LiboV) or not annotated (Solomon Islands) in the other isolates.

Complete genome sequences of OrNV have been previously determined from the Malaysian isolate OrNV-Ma07^13^, OrNV-Solomon Islands^14^ and an Indonesian isolate, OrNV-LiboV (GenBank accession no. MT150137). There are also genome-length contigs reported by Etebari, Parry, et al.^15^ that had been assembled from several OrNV transcriptomes, but the consensus sequences for these assemblies have not been confirmed by sequencing of viral DNA and are not publicly available in GenBank. Genome sizes of the OrNV-X2B and OrNV-Palau1 isolates are 125,905 bp and 126,039 bp (GenBank accession no. MW298153 and MW298154), respectively, which are similar to those reported for OrNV-Solomon Islands and OrNV-LiboV (125,917 bp and 125,846 bp, respectively), but are approximately 1.6 kbp shorter than reported for OrNV-Ma07 (127,615 bp) (Table 2). The difference in genome sizes between OrNV-Ma07 and the other OrNV isolates can be attributed partly to an approximately 740 bp stretch of DNA in OrNV-Ma07, containing ORF91 of this isolate, which is absent from the genomes of the other isolates (Fig. 4); and partly to an inversion of a region of the genome bound by OrNV-Ma07 ORFs 128 and 136 which resulted in the loss of ORFs 129 and 130/135 (Fig. 5). This inversion was previously identified in the analysis of the Solomon Islands isolate^14^, and appears to be a consequence of recombination between two regions in the Ma07 isolate containing ORFs 129 and 130 and ORFs 135 and 136, respectively, that are inverted duplicates of each other (Fig. 5).

**Table 2:** Isolates of Oryctes rhinoceros nudivirus (OrNV) with completely sequenced genomes.

Isolate	Origin	GenBank ID	%GC	Size, bp	Annotated ORFs	Notes and references
OrNV-Ma07	Malaysia	Eu747721 (NC_011588)	41.63	127,615	139	Representative isolate; Wang et al. 2011
OrNV-Solomon Island	Solomon Islands	MN623374	41.65	125,917	130	Etebari et al. 2020
OrNV-LiboV	Indonesia	MT150137	41.71	125,846	123	Unpublished (submitted to GenBank 03-MAR-2020)
OrNV-X2B	Palawan, Phillipines	MW298153	41.65	125,905	132	This study
OrNV-Palau1	Melekeok, Palau	MW298154	41.66	126,039	129	This study

Pairwise alignments of the X2B and Palau1 genome nucleotide sequences with each other and with the sequences of the other OrNV isolates in Table S1 yielded sequence identities ranging from 98.3 to 99.9%. Alignments with the Ma07 sequence were characterized by relatively low identities—98.3% and 98.9% with Palau1 and X2B, respectively—and a large number of gaps and mismatches due to the inversion described above and pictured in Fig. 5. In contrast, alignments involving the other isolates yielded sequence identities of at least 99.6%.

Homologs for most of the ORFs present in the Ma07 isolate are also annotated in genomes of the other isolates (Supplementary Table S1 and S2). Ma07 ORFs 82 and 91 are not in the Solomon Islands, X2B, or Palau1 genomes. ORFs 32 and 50 are missing from the Solomon Islands and X2B isolates, while ORFs 70 and 85 are missing from the X2B and Palau1 isolates. The Palau1 isolate is also missing ORFs 31, 49, 67, and 99. In most cases, the missing ORFs were not annotated due to substitutions or short frame-shifting insertions and deletions in the sequence that created premature stop codons. A 1-nt insertion in both the Palau1 and X2B isolates resulted in a fusion of Ma07 ORFs 83 and 84 into a single ORF. A previously unidentified ORF, detected by both the fgenesV0 and the VGAS ORF-finding programs, was annotated in the X2B and Palau1 genomes. This ORF lies between the homologs of Ma07 ORFs 130 and 136 (Fig. 5, red ORF) and encodes a predicted 76-amino acid polypeptide with no significant similarity to other sequences detectable by either BLASTp or HHpred queries. While homologs of this unique ORF are not present in the Ma07 or LiboV genome sequences, it is conserved with 100% sequence identity in the Solomon Islands isolate.

**Figure 6:**
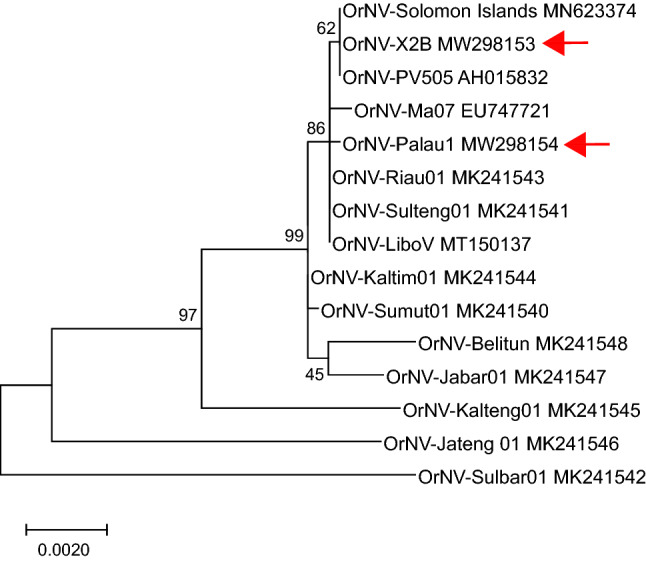
Relationships among OrNV isolates inferred from alignment of DNA polymerase nucleotide sequences. A midpoint-rooted phylogram inferred by maximum likelihood is shown, with bootstrap support (%) indicated. The positions of sequences from OrNV-X2B and OrNV-Palau1 are indicated by red arrows in the tree.

Pairwise protein BLAST analyses with ORFs conserved among isolates Ma07, Solomon Islands, X2B, and Palau1 yielded mean amino acid sequence identities ranging from 98.54% (Ma07 × Palau1) to 99.17% (X2B × SI), with median sequence identities of 100% for all comparisons. Homologs of Ma07 ORFs 66, 68, and 81 exhibited sequence identities that were significantly lower than average, due to frameshifting mutations in these ORFs. Phylogenetic inference from OrNV DNA polymerase nucleotide alignments placed the X2B isolate, which derives from a non-G haplotype host, in a clade containing the Solomon Islands isolate from a G-type host and isolate PV505 from the Philippines (original haplotype unknown) (Fig. 6)^15,16^. This clade was part of a larger clade containing the Palau1 and LiboV isolates, as well as DNA polymerase sequences from two of a set of nine Indonesian isolates^17^.

## Discussion

A majority of the field-captured *O. rhinoceros* adults (60%) were CRB-G, and 77.5% of both haplotypes were OrNV-positive by PCR detection (Table 1) in Palau, which is consistent with a previous report^9^. A high prevalence of OrNV also has been observed in the field-trapped CRB population in Malaysia (65%)^18^, and in Fiji (62%)^9^ where OrNV had been applied for control over a long period of time^2^. In Palau, OrNV was introduced in 1970 and 1982, but the strain used for control was not recorded^19^. A high prevalence of OrNV may be due to the persistence of previously introduced viruses. Because there was no significant difference between the prevalence of OrNV in the two haplotypes, CRB-G in the Palauan population appears to be as susceptible to OrNV as CRB-S. Consistently, a high prevalence in CRB-G (from 64 to 100%) was also detected from traps in Solomon Islands, New Caledonia and Philippines^10^. On the other hand, the virus was not detected from CRB-G in Guam and Hawaii^9^. Because *COI* gene is used as haplotype marker^9^, but is encoded in mitochondrial DNA and maternally transmitted, the genetic regions responsible for susceptibility of *O. rhinoceros* to OrNV are more likely located in nuclear genes rather than mitochondrial genes. In Palau where CRB-G and CRB-S coexist, if the two haplotypes mate with each other, their offspring would have nuclear genomes of both parents, and a maternal mitochondrial genome. Thus, nuclear genes of Palauan CRB-G may be phylogenetically different from that in Guam and Hawaii. It has been reported that the Palauan CRB has a different genetic background from that of Guam and Hawaii by phylogenic analysis of nuclear genomic DNA using ddRAD-seq from various areas^20^. Because it was known that susceptibility to OrNV varied depending on the combination of virus isolates and hosts^21^, susceptibility of the Palauan CRB-G to OrNV may be different from CRB-G in Guam. Further studies are needed to compare the relatively susceptibilities of CRB-G from Palau and Guam to OrNV.

The tissues of OrNV PCR-positive samples of CRB-G in Palau were observed using TEM. Nudivirus-like particles were observed in the midgut and fat body in field-captured Palauan adults CRB (Fig. 2). The infectivity of the Palauan virus, which was extracted from tissues of infected adults collected from Palau, was tested by hemocoel injection into second instar *O. rhinoceros* originating from Japan. Consequently, 7 of 8 larvae were dead with swollen or prolapsed guts as described previously^6^. From these samples, expression of viral mRNA (*p74,* structural gene) and virus particles were detected by RT-PCR and TEM, respectively (Fig. 2). Therefore, pheromone trap-captured adults were not killed by infection of OrNV before examination (as defined as sublethal infection) but carry potentially lethal active virus against CRB larvae. Thus, in Palau, adult beetles, which were sublethally infected, might fly around with the virus particles produced in its host, and spread the virus as virus carrier and spreader.

In this study, a new cell culture system for OrNV replication was demonstrated using AnCu35 cells, which were established from *Anomala cuprea* embryo tissue. AnCu35 cells inoculated with OrNV-X2B showed CPE at 5–6 dpi (Fig. S1), suggesting OrNV is able to infect and replicate within AnCu35 cells. Previously only DSIR-Ha-1179 cells, established from *Heteronychus arator (Subfamily: Dynastinae)* embryo tissue, were known to be permissive for OrNV replication^12^. Similarly, *Allomyrina dichotoma (Subfamily: Dynastinae)* can be infected with OrNV^22^, but *A. cuprea* belongs to the subfamily Rutelinae. It suggests that the host range of OrNV may be wider than expected. Research to investigate this possibility with insects is needed.

Although different life stages were studied, the fact that CRB-G larvae from Japan could be infected with OrNV by intrahemocoelic injection was consistent with a previous report that CRB-G adults from Guam were infected with OrNV by injection^9^. The pathology caused by the OrNV-Palau1 isolate in Japanese-sourced CRB larvae was examined and compared to that of the OrNV-X2B isolate. TEM analysis revealed OrNV particles were produced following treatment with OrNV-Palau1, which was also observed for the OrNV-X2B virus isolate treatment. Steady-state levels of genomes produced in larvae inoculated with OrNV-Palau1 was less than that with OrNV-X2B on 9 dpi. This is the first study monitoring viral DNA multiplication of OrNV in CRB larvae by qPCR. In addition, OrNV-Palau1 killed CRB larvae two days later than X2B. Thus, the OrNV strain isolated from Palau adults was less virulent against CRB larvae in Japan than X2B, an isolate that is commonly used in the Pacific region for CRB control. While potentially less virulent, the high detection rate of adults with sub-lethal infections observed in Palau may be due to the possibility that OrNV-Palau1 may be more infective, and therefore the virus could be more easily transmitted to healthy adults. Further research is needed to demonstrate oral infectivity of OrNV and transmission of virus between Palauan-sourced CRB adults, but the susceptibility of Japanese-sourced larvae to OrNV provides an opportunity to carry out further comparative lab and field research to characterize differences between OrNV isolates.

Sequence determination of the OrNV-Palau1 and -X2B isolate genomes, and comparison with sequences from other OrNV isolates, confirmed that OrNV genomes are highly conserved with few differences in structure or ORF content. Although OrNV-Ma07 is the representative isolate of *species Oryctes rhinoceros nudivirus*, there are two large rearrangements –covering an approximately 740 bp region containing ORF91 (Fig. 5), and an inversion of a larger region containing five ORFs—that are unique to the Ma07 isolate. Phylogenetic relationships inferred from an alignment of OrNV DNA polymerase sequences grouped isolates from CRB-G hosts (Palau, Solomon Islands) with isolates from non-CRB-G hosts (Ma07, X2B). Further investigation of other OrNV isolates will help to identify genomic variants that correlate with the ability to infect and replicate within CRB hosts.

CRB uses decaying organic matter (particularly coconut) as breeding sites where eggs are laid for larvae to feed^7^. However, OrNV infectivity degrades to under 1% in soil within a week^23^, thus for effective transmission of OrNV, rapid transmission among CRB and a minimum population density of the host beetle would be needed. Given this, it is useful to not only focus on OrNV isolates with strong lethal activity, but also take into consideration the sublethal effect within the context of pest control campaign. It is known that sublethal infection by virus can decrease fitness of its insect host. For example, it was reported that 80–100% of *Malacosoma californicum pluviale* are sublethally infected with Malacosoma californicum pluviale nucleopolyhedrovirus in Canada, where the fecundity of the host population decreased as compared due to the sublethal viral infection^24^. For CRB, it has been reported that the lifespan and fecundity of adults infected with OrNV significantly decreases, and that feeding activity, flying and mating are negatively influenced in infected adults^25,26^. In Palau, the widespread presence of OrNV infected adults in field-trapped CRB suggests the possibility that reduced virulence or sublethal effects may play a role in the CRB-OrNV pathology observed there. Further field and genetic investigations will be needed to better define and understand the nature of the interactions between the OrNV-Palau1 pathogen, the current Palauan CRB host population, and the effect on the palm damage in Palau.

## Methods

### Insects and virus

*Oryctes rhinoceros* was collected from Amami, Kagoshima, Japan in 2017 and Ishigaki, Okinawa, Japan in 2018. The insects were brought back to the lab in Tokyo and maintained in a moisture mushroom mat substrate (Mushroom Mat, Tsukiyono Kinokoen, Japan) which was also served as food for larvae. The temperature was held at 25–30 °C with a 16-h light / 8-h dark photoperiod. To collect eggs, 2 or 3 female adults were put in a plastic case containing a moisture mushroom mat substrate with a male adult beetle. The insect jelly (Dorcus Jelly, Fujikon, Japan) was provided *ad libitum* as food for adults. After 2 weeks, we collected eggs, and about 10 eggs were placed in a plastic cup with a moisture mushroom mat substrate until hatched larvae developed to the second instar. This strain was used in all bioassays in this study. All Japanese *O. rhinoceros* were confirmed as CRB-G.

The OrNV-X2B isolate used in this study was originally isolated from Philippine CRB and obtained from AgResearch in New Zealand.

### Cell cultures

FRI-AnCu-35 (AnCu35) cells were obtained from Genebank of NARO (Tsukuba, Japan)^27^. This continuous cell line was developed from embryos of the cupreous chafer, *Anomala cuprea* (*Coleptera: Scarabaeidae*). The cells were maintained as adherent cultures in 25 cm^2^ tissue culture flasks (Falcon, Corning, USA) at 25 °C in 5 ml of 10% Fetal Bovine Serum (Gibco, Thermo Fisher Scientific, USA) supplemented Grace’s insect medium (Gibco). Cells were passaged in the above culture medium until the cell monolayer reached 70% confluence.

### DNA extraction and identification of haplotypes in Palauan population

CRB specimens were collected in Palau using pheromone traps containing ethyl 4-methyloctanoate (ChemTica Internacional, Costa Rica). Adults were dissected to collect midgut and gut tissues to avoid cross contamination between dissection of individuals, which were immediately soaked into 0.1 μg/ml gentamicin solution to prevent bacterial contamination during transportation at room temperature. Specimens were stored at − 30 °C after arrival to Tokyo. The tissues were homogenized in cell lysis solution (10 mM Tris–HCl, 100 mM EDTA, 1% SDS, pH 8.0) using pestles in 1.5 ml microcentrifuge tubes. Homogenates were centrifuged at 12,000× g for 5 min at 4 °C. Proteinase K (200 µg/ml final concentration) (Nippon Gene Co. Ltd., Japan) was added to the supernatant and incubated at 50 °C for 5 h. To remove contaminating RNA, RNase A solution (100 µg/ml final concentration) (Nippon Gene Co. Ltd.) was added. After a 30 min incubation at 37 °C, the mixture was placed on ice and supplemented with 200 μl of Protein Precipitation Solution (Qiagen, Germany), and then centrifuged at 17,000× g for 15 min at 4 °C. The supernatant was isopropanol-precipitated, pelleted by centrifugation, and washed with 70% ethanol. Finally, precipitated DNA was dissolved in distilled MilliQ water. The concentrations of each DNA solution were measured by using NanoVue Plus (GE Healthcare, Buckinghamshire, England, UK). The sample DNA was diluted to 10 ng/μl and used for PCR. The following primer pair was used to amplify a 523 bp fragment of the COI gene: C1-J-1718Oryctes (5′-GGAGGTTTCGGAAATTGACTTGTTCC-3′) and C1-N-2191Oryctes (5′-CCAGGTAGAATTAAAATRTATACCTC-3′)^9^. Each 10 μl PCR reaction contained: 5 μl Emerald Amp (Takara, Japan), 0.3 μl forward primer (10 μM), 0.3 μl reverse primer (10 μM), 3.4 μl Milli-Q water (Merck Millipore, USA), and 1 µl template DNA. PCR amplifications were performed in a Life ECO thermocycler (Bioer Technology, China) with a cycling profile of 35 cycles of 94 °C denaturation (30 s), 50 °C annealing (45 s), 72 °C extension (1 min) with an initial denaturation of 3 min at 94 °C and a final extension of 5 min at 72 °C. A 5 μl aliquot of each PCR amplicon was checked by agarose gel electrophoresis (1.5%, 1 × TBE), stained with Midori green (Nippon Genetics, Japan) and fluorescence visualized over UV light. Photographs were recorded using an E-BOX-VX2 /20 M (E & M, Japan).

For direct sequencing, the PCR products were purified using a QIAquick PCR Purification Kit (Qiagen). The purified DNA was sequenced using BigDye Terminator Kit ver. 3.1 (Applied Biosystems, USA) and performed by the 3700 DNA analyzer (Applied Biosystems). The obtained sequences were analyzed using MEGA X software^28^ and the G haplotype was identified by the presence of the (A→G) point mutation in the *COI* region as previously described^9^.

### Virus detection in Palauan population

Using the same samples as above, virus detection was carried out by PCR. The following primer pair was used to amplify a 944 bp fragment of the *OrNV-gp054* gene (GrBNV-gp83-like protein): OrNV15a (5′-ATTACGTCGTAGAGGCAATC-3′) and OrNV15b (5′-ATGATCGATTCGTCTATGG-3′)^29^. PCR amplifications were performed as above.

Transmission electron microscopy (TEM) was also used for detection of OrNV within a subset of PCR positive CRB tissue samples. After washing in phosphate-buffered saline (PBS), midgut and fat body samples of Palauan CRB adults from Melekeok and Aimeliik (respectively; two each), were subjected to following resin fixation as described previously^30^: tissues were fixed in 5% glutaraldehyde for 1 h, rinsed 4 times with Millonig’s phosphate buffer (0.18% NaH_2_PO_4_ × H_2_O, 2.33% Na_2_HPO_4_ × 7H_2_O, 0.5% NaCl, pH 7.4), post-fixed and stained in 1% OsO_4_ for 2 h and dehydrated in an ethanol series. Following the final dehydration step, the ethanol was replaced by QY-1 (Nisshin EM, Tokyo), and the tissues were embedded in epoxy resin comprising 47% TAAB EPON812, 19% DDSA, 32% MNA and 2% DMP30 (Nisshin EM, Tokyo). Then, they were cut into 70 nm thick sections with a diamond knife on an Ultracut N ultramicrotome (Leica, Vienna, Austria), attached to grids and observed using TEM (JEM-1400Plus, JEOL, Japan).

### Isolation of OrNV from Palauan samples and infectivity to Japanese CRB larvae

Virus isolation was carried out using a modification of a method previously described^23^. The frozen tissues of two virus positive CRB-G from Melekeok were washed with PBS twice, and after grounding with 1 ml PBS by pestles, centrifuged at 6,000 g × 5 min at 4 °C. The supernatant was filtered by 0.45 µm pore sized filter (Merck, USA) and transferred to a 1.5 ml ultracentrifuge tube in a clean bench. Virus was pelleted by centrifugation at 4 °C, 98,600 g for 30 min using a TLA55 rotor. After separation, the supernatant was discarded and the pellet was suspended in 500 μl of PBS and designated as “virus solution”. A portion of this solution (30 µl/larva) was intrahemocoelically injected into 82nd instar CRB to evaluate its infectivity. This experiment had no biological replicates due to the very small amount of inoculum available. Intrahemocoelically injected larvae were reared in the insect rearing mat at 25 °C for two weeks. Following death, larval cadavers were immediately dissected to collect midgut for following RNA extraction to detect expression of a viral gene, and electron microscopy observation. Total RNA was extracted from larval tissue samples using ISOGEN (Nippon Gene Co. Ltd., Tokyo, Japan), as described in the manufactural protocol. The total RNA samples were treated with RNAse-free recombinant DNAse I (TaKaRa, Japan) to remove the contaminating DNAs. The DNAse I treated total RNA samples (approximately 100 ng/µl) were used as templates for cDNA synthesis using a TaKaRa RNA PCR Kit (AMV) ver. 3.0 (TaKaRa, Japan). PCR reactions were conducted as above using OrNV15a and b primers (detects gene GrBNV-*gp83*-like gene). This experiment was conducted in triplicate.

### Inoculum preparation using FRI-AnCu-35 cells

OrNV isolates were propagated using the FRI-AnCu-35 (AnCu35) cell line for further analyses following methods previously described for the DSIR-Ha-1179 cell line system^9,12^. AnCu35 was a Coleopteran cell line readily available in Japan, and was inoculated with the Palau OrNV solution prepared above and the OrNV-X2B isolate which was provided by AgResearch, New Zealand. When the cell culture reached 25% confluency, a 100 µl aliquot of virus solution was inoculated and incubated at 25 °C. The virus-treated cells were observed by optical microscope.

Quantification of viral copy number using qPCR was conducted as follows. To measure the amount of OrNV virus produced by the AnCu35 cell line, DNA was extracted as described above for tissue samples from 1.5 ml of the virus treated cell’s suspension at 10 dpi (3 suspensions per each virus isolate). The extracted DNA was subjected to quantitative PCR (qPCR) following previously described methods^31^. The primer pair for qPCR was designed from the genome sequence of the *P74* homolog of OrNV, a viral structural protein that is conserved widely among nudiviruses, polydnaviruses and baculoviruses^32^, to amplify a region of 82 bp of OrNV-X2B-*gp120* (OrNV-*p74*_f2026: 5′-ATCGCCGGTGTGTTTATGG-3′, OrNV-*p74*_r2107: 5′-AGAGGGCTAACGCTACGAC-3′). The qPCR reaction was performed by using Step One Plus Real-Time PCR System (Life Technologies, USA). The reaction mixture contained 10 ng of template DNA, 5 µl of FastStart Universal SYBR Green Master Mix (ROX) (Roche, Switzerland), 0.3 µl forward primer (10 µM), 0.3 µl reverse primer (10 µM), and 3.4 µl Milli-Q water. The qPCR cycle condition was as follows: 95 °C 10 min; 40 cycle of 95 °C 15 s, 60 °C 1 min. At the end of the cycles, a dissociation curve analysis of the amplified product was performed as follows: 95 °C 15 s, 60 °C 1 min, 95 °C 15 s. The Ct value of each sample DNA was measured twice using two wells as technical replicates. The quantity of the viral genome (ng) in each sample was calculated from a standard curve generated from 29.7 to 29.7 × 10^–5^ ng of purified PCR amplicon from the OrNV *P74* gene. The viral copies in 1 ng of sample DNA was estimated from the molecular weight of qPCR target region (*p74*). The virus titer was determined from average copy numbers of three virus suspensions as follows. The *p74* qPCR amplicon was 83 bp, and the molecular weight of the amplicon was calculated as the length of dsDNA (83 bp) × 330 daltons × 2 nt/bp = 54,780 daltons (g/mol). DNA weight of 1 copy of virus genome was calculated as 54,780 g/mol/Avogadro constant (6.023 × 10^23^ molecules/mol) = 9.095 × 10^–20^ g/ molecule. Amplicons of the above region was purified by QIA quick PCR purification kit (Qiagen) and 29.7 ng/ul of DNA was obtained for use as a quantification standard. This is equivalent to 3.266 × 10^11^ copies of *p74* gene (because the amplicon is 9.095 × 10^–20^ g/copy). Based on qPCR using the serial dilutions (× 10 – 10^5^) of the standard DNA prepared above, Ct values were examined by each concentration of viral DNA. Ct-value = − 3.3112x – 1.4219 (x: diluton factor of 10^x^). Accordingly, copy number of *p74* = 3.266 × 10^11+x^. Viral copy number (copy number of *p74* genes) was calculated from Ct-value from the above formula.

### Viral replication in CRB larvae by time course and killing speed

Field collected CRB-G larvae from Japan were inoculated with the OrNV-Palau1 and -X2B isolates to examine establishment of infection over time using qPCR. The inoculum was prepared from supernatant collected from OrNV infected AnCu35 cell cultures at 10 dpi, passed through a 0.45 µm filter, and preserved at 4 °C until use.

Second instar CRB was inoculated intrahemocoelically with 30 μl of the virus solution prepared from cell-culture per larva using a microinjector (Kiya Kogyo Seisakusho, Japan) fitted with a micro-syringe (Ito Seisakusho, Japan). The virus doses of OrNV-Palau1 and -X2B strains used for inoculation were confirmed to be comparable by absolute quantification using the above qPCR method (Palau1: 3.1 × 10^5^ copies/ng, X2B: 3.3 × 10^5^copies/ng; the mean titer of 3 DNA templates, respectively). As a mock treatment, CRB was injected with 30 µl PBS. The inoculated larvae were kept individually in plastic containers with a rearing mat in a 25 °C incubator. The samples were collected at 3, 6, and 9 dpi (25–30 larvae per time point) into 15 ml tubes and stored at − 30 °C until the DNA was extracted as above. Total DNA was extracted from whole, individual larvae which were dissected to remove midgut contents to prevent interference to Taq polymerase, and subjected to qPCR as above. Changes in viral copy number within the same virus strain over time were analyzed by one-way, nonparametric Steel–Dwass tests using JMP@ 9.0.0 software (SAS Institute, Cary, NC). Differences in virus copy number between strains were analyzed in the same way, but to correct for errors in the test values due to multiple comparisons, Bonferroni's correction was used to set the α-value for the test at 0.008333. Ten larvae were inoculated and examined per each treatment-time point with three replications.

To estimate killing speed, CRB-G larvae from Japan were inoculated with the OrNV-Palau1 and -X2B isolates as described previously. Intrahemocoelically inoculated larvae were reared individually in plastic containers with a rearing mat in a 25 °C incubator. Mortality of inoculated larvae were observed every day. Forty larvae were examined in a replicate with three replications carried out for virus treatments (total 120 larvae). The mock PBS inoculation treatment was done only once (total 37 larvae).

### Genome sequencing

Genome sequencing of the OrNV-Palau1 isolate and X2B isolate was conducted. For obtaining high quality DNA, virus particles were purified, from 3 mL of AnCu35 culture supernatant collected six days after inoculation with OrNV. Virus containing supernatant was transferred to Ultra-Clear polyallomer tubes (Beckman Coulter, USA) with a 20–50% (w/w) sucrose density gradient and subjected to ultracentrifugation at 72,100 g, 4 °C, for 1 h. After ultracentrifugation, the white virus band was collected in a 1.5 ml tube. The solution was then subjected to ultracentrifugation at 110,000 g, 4 °C for 1 h to precipitate the viral particles^33^. Then, DNA was extracted from purified OrNV virions as described above. For the sequencing analysis, DNA libraries were prepared using the Nextera XT DNA Library Prep Kit (Illumina, USA). Amplified libraries were sequenced on Illumina HiSeq 2500 instrument using paired-end 2 × 150 bp chemistry which was performed by Novogene (Beijing, China). Contigs of each strain from NGS reads were generated by assembly using Unicycler (version 0.4.8)^34^. The gaps between contigs were further closed with Sanger sequences obtained by PCR direct sequencing using appropriate specific primers, and the sequence was aligned by minimap2 (version 2.17)^35^. The assembly and sequences of contigs were also confirmed by mapping to the OrNV isolate Solomon Islands genome sequence (GenBank accession no. MN623374.1) with NGS reads and Sanger sequences using minimap2. The mapped reads (SAM files) were converted to BAM format using SAMtools (version 1.10)^36^. After the sorting and indexing of BAM files, the consensus sequences were generated using bcftools (version 1.10.2)^37^.

ORFs of at least 50 codons in size that possessed significant amino acid sequence similarity with ORFs from OrNV-Ma07 were identified with Lasergene GeneQuest (DNAStar, v. 17) and BLASTp. ORFs with no significant matches to other sequences also were selected for annotation if (a) they did not overlap a larger ORF by > 75 bp, and (b) they were predicted to be protein-encoding by both the fgenesV0 (http://www.softberry.com/berry.phtml?topic=index&group=programs&subgroup=gfindv) and Vgas^38^ programs.

OrNV genome sequences were compared by pairwise alignment using the Martinez/Needleman-Wunsch method as implemented in Lasergene MegAlign 15. Pairwise sequence identities were determined from these alignments as previously described^39^. Differences in ORF content and distribution of selected OrNV genomic regions were visualized with Mauve version 20150226^40^.

### Phylogenetic inference

To infer the relationships among OrNV isolates on the basis of nucleotide sequence alignments, the DNA polymerase ORFs of completely sequenced isolates (Table 2), OrNV-PV505^16^, and a set of nine isolates from Indonesia^17^ were aligned by MUSCLE as implemented in Lasergene MegAlign Pro v. 17 (DNAStar). Phylogeny was inferred by maximum likelihood using MEGA X^28^ with the Tamura-Nei (TN93) model^41^, with ambiguous data eliminated prior to analysis. Tree reliability was evaluated by bootstrap with 500 replicates.

## Supplementary Information

Supplementary Figure S1.

Supplementary Table S1.

Supplementary Table S2.
