# Supplementary material for: Confirmation of Oryctes rhinoceros nudivirus infections in G-haplotype coconut rhinoceros beetles (Oryctes rhinoceros) from Palauan PCR-positive populations
Source: Sci Rep. 2021 Sep 20;11:18820. doi: 10.1038/s41598-021-97426-w (PMC8452681; doi:10.1038/s41598-021-97426-w)
Supplement: Supplementary file 1 — Supplementary Figure S1. [file 41598_2021_97426_MOESM1_ESM.pdf]

(A) Virus treated AnCu35 cells

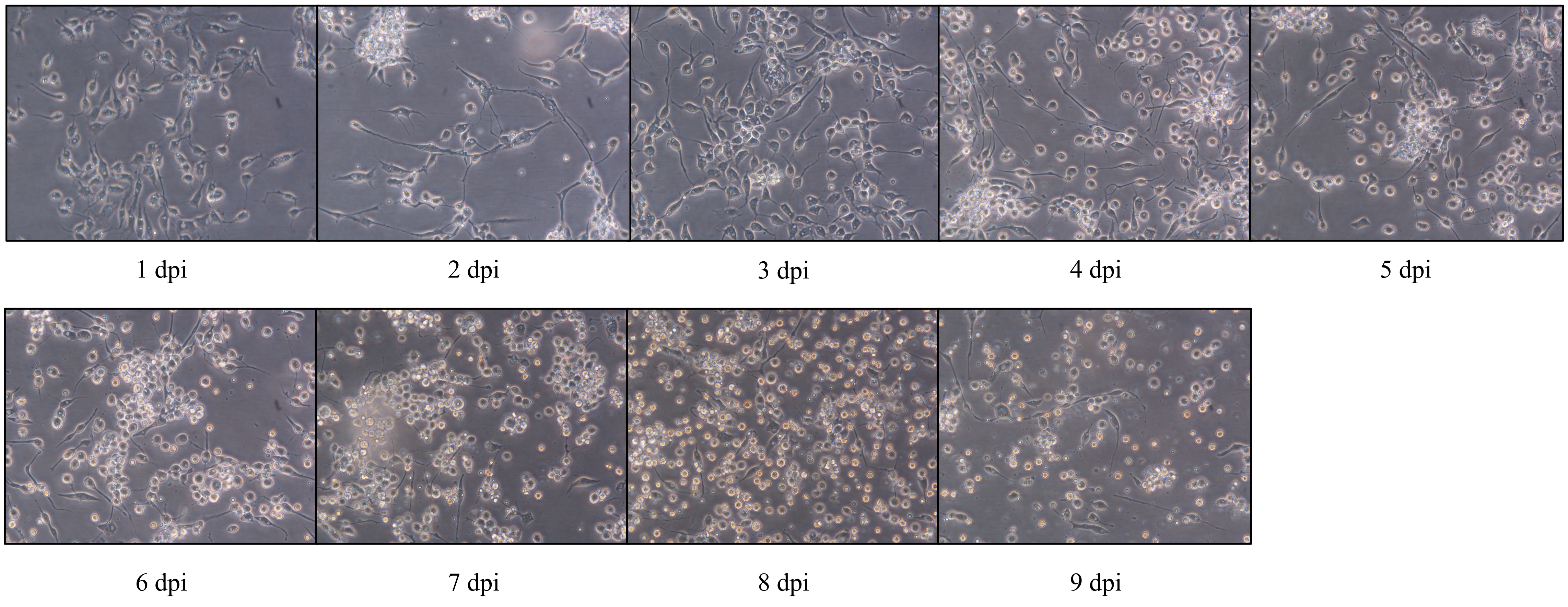

(B) Untreated AnCu35 cells

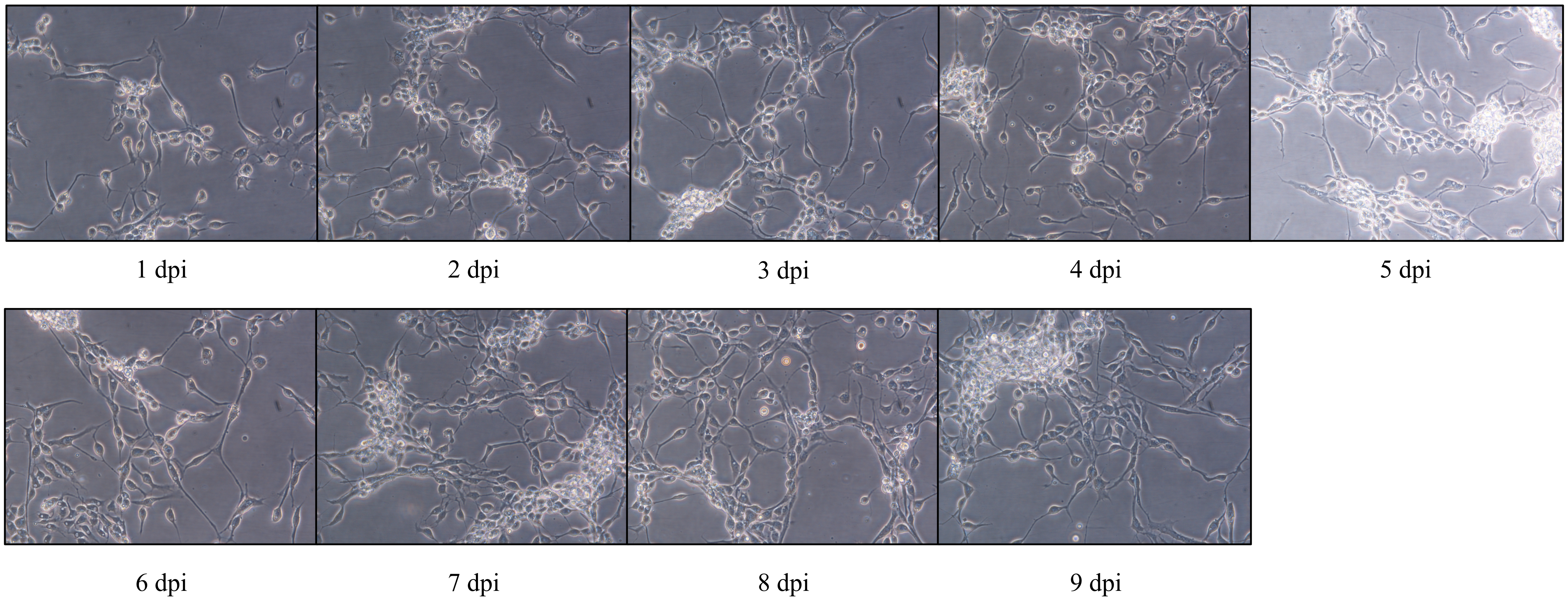

Fig.S1. Optical micrographs of FRI-AnCu-35 (AnCu35) cells inoculated with OrNV X2B isolate.
AnCu35 cells inoculated with OrNV X2B (A) were observed every day until 9 dpi and untreated healthy cells (B). Magnifications were all ×400.
