# Supplementary material for: Confirmation of Oryctes rhinoceros nudivirus infections in G-haplotype coconut rhinoceros beetles (Oryctes rhinoceros) from Palauan PCR-positive populations
Source: Sci Rep. 2021 Sep 20;11:18820. doi: 10.1038/s41598-021-97426-w (PMC8452681; doi:10.1038/s41598-021-97426-w)
Supplement: Supplementary file 2 — Supplementary Table S1. [file 41598_2021_97426_MOESM2_ESM.pdf]

Table S1. Open reading frames of isolates OrNV-Palau1

OrNV-Palau1 ORFs						BLAST best match					
						OrNV-X2B		OrNV-Ma07		OrNV-SI	
**ORF No.**	**Annotation**	**Start**	**End**	**aa**	**Direction**	ORF No.	%Identity	ORF No.	%Identity	ORF No.	%Identity
1	dnapol B	1	3843	1281	forward	1	99.92	1	100	1	99.84
2	putative trypsin-like serine protease	3898	4917	340	forward	2	100	2	100	2	100
3	GrBNV_gp13-like protein	4928	6043	371	forward	3	100	3	100	3	99.73
4	Ac81-like protein	6067	6564	497	forward	4	99.37	4	100	4	100
5	arenavirus nucleocapsid C-terminal domain-like protein	7152	6580	191	reverse	5	99.47	5	100	5	99.47
6	thymidylate synthase/pyrimidine hydroxymethylase-like protein	8164	7199	322	reverse	6	99.69	6	99.69	6	99.69
7	putative calcineurin-like phosphoesterase	9042	8209	277	reverse	7	99.66	7	99.64	7	99.64
8	hypothetical protein	9301	9134	55	forward	8	100	8	100	8	100
9	NeseNPVORF52-like protein	9300	9965	222	reverse	9	100	9	99.55	9	100
10	hypothetical protein	10194	9994	67	forward	10	100	10	92.31	10	90.77
11	mitochondrial carrier protein-like protein	10331	11119	262	reverse	11	100	11	100	11	99.62
12	odv-e66	12930	11149	594	reverse	12	99.66	12	99.66	12	99.66
13	hypothetical protein	13584	12982	201	reverse	13	100	13	100	13	100
14	hypothetical protein	13796	13587	69	reverse	14	100	14	100	14	100
15	vp39 protein	14469	13717	251	forward	15	100	15	100	15	100
16	FEN-1	14537	15883	449	reverse	16	100	16	99.78	16	100
17	PIF-2	17040	15916	375	forward	17	100	17	100	17	100
18	GrBNV_gp67-like protein	17181	18290	370	forward	18	99.73	18	99.19	18	99.73
19	Ac146-like protein	18312	18623	104	forward	19	100	19	99.03	19	100
20	P47	18535	19509	324	reverse	20	100	20	100	20	100
21	hypothetical protein	20604	19579	341	forward	21	99.71	21	99.71	21	99.71
22	GrBNV_gp72-like protein	20603	21334	244	reverse	22	99.18	22	100	22	99.18
23	guanylate kinase-like protein	22218	21460	253	reverse	23	100	23	100	23	100
24	GrBNV_gp75-like protein	22839	22258	193	forward	24	100	24	100	24	100
25	GrBNV_gp76-like protein	22838	24496	553	forward	25	99.64	25	99.64	25	99.46
26	hypothetical protein	24557	25720	388	reverse	26	100	26	100	26	100
27	GrBNV_gp78-like protein	26961	25756	402	forward	27	99.75	27	99.75	27	99.75
28	hypothetical protein	26992	27828	279	reverse	28	99.64	28	99.64	28	99.64
29	GrBNV_gp81-like protein	28619	27840	259	reverse	29	100	29	100	29	100
30	VLF-1	28486	30612	709	forward	30	99.86	30	99.44	30	99.86
31	hypothetical protein	31227	31382	51	forward			32	94.87		
32	PIF-4	32505	31738	256	forward	32	99.61	33	100	32	100
33	DNA helicase	32620	36342	1241	reverse	33	99.92	34	99.92	33	99.76
34	hypothetical protein	36715	36422	98	reverse	34	100	35	100	34	100
35	hypothetical protein	37153	36731	141	reverse	35	99.29	36	100	35	99.29
36	PP78/83	38140	37214	309	reverse	36	99.35	37	99.35	36	99.35
37	hypothetical protein	38453	38172	93	reverse	37	100	38	100	37	100
38	GrBNV_gp93-like protein	39107	38538	190	forward	38	100	39	100	38	100
39	GrBNV_gp94-like protein	39081	39479	132	forward	39	100	40	100	39	100
40	GrBNV_gp95-like protein	39451	39792	114	forward	40	100	41	99.12	40	100
41	LEF-4	39768	40973	402	forward	41	100	42	99.75	41	100
42	hypothetical protein	41120	40965	51	reverse	42	100	43	100	42	100
43	GrBNV_gp97-like protein	41082	41672	197	reverse	43	100	44	100	43	100
44	GrBNV_gp23-like protein	42656	41685	324	reverse	44	100	45	100	44	100
45	GrBNV_gp22-like protein	44280	42709	524	reverse	45	99.81	46	99.81	45	99.81
46	GrBNV_gp19-like protein	45474	44482	331	reverse	46	100	47	100	46	100
47	hypothetical protein	45903	45742	54	forward	47	96	48	96.23	47	96.23
48	hypothetical protein	46312	46467	51	forward			50	100		
49	RR1	46634	48850	739	reverse	49	100	51	99.73	49	99.86
50	LEF-5	49149	48913	79	forward	50	100	52	100	50	100
51	GrBNV_gp84-like protein	49840	49193	215	forward	51	100	53	100	51	100
52	GrBNV_gp83-like protein	50494	51813	439	reverse	52	100	54	99.77	52	100
53	hypothetical protein	51994	51797	65	reverse	53	100	55	100	53	100
54	hypothetical protein	53524	52175	450	forward	54	99.56	56	99.55	54	99.56
55	patatin-like phospholipase-like protein	53657	54766	370	reverse	55	99.73	57	100	55	99.73
56	HZV_115-like/thymidylate kinase-like protein	55434	54805	210	reverse	56	99.52	58	99.52	56	99.52
57	LEF-3	56037	55519	172	reverse	57	100	59	100	57	100
58	PIF-1	57620	56145	492	forward	58	99.8	60	100	58	100
59	GrBNV_gp51-like protein	57705	58046	114	forward	59	97.39	61	98.25	59	100
60	hypothetical protein	58411	58145	89	forward	60	100	62	100	60	100
61	hypothetical protein	58365	58586	73	forward	61	100	63	100	61	100
62	LEF-8	61380	58612	922	reverse	62	100	64	100	62	100
63	hypothetical protein	61848	61666	61	reverse	63	100	65	100	63	100
64	hypothetical protein	62368	62123	81	reverse	64	70	66	72.13		
65	hypothetical protein	62918	62562	118	reverse	66	99.12	68	77.78		
66	FIC-like protein	63215	63877	220	forward	67	100	69	100	65	100
67	hypothetical protein	64195	64875	227	forward	68	99.12	71	99.56	67	99.12
68	PIF-6	65467	65057	137	reverse	69	100	72	100	68	100
69	hypothetical protein	65743	66528	261	forward	70	99.28	73	100	69	99.23
70	densovirus NS3-like protein	66656	67528	291	forward	71	100	74	98.97	70	100
71	integrase/recombinase-like protein	68669	67575	365	reverse	72	99.73	75	99.73	71	99.73
72	GrBNV_gp58-like protein	68716	68871	52	forward	73	100	76	100	72	100
73	semaphorin-like protein	68964	69332	123	forward	74	100	77	100	73	100
74	semaphorin-like protein	69251	70645	464	forward	75	100	78	100	74	100
75	GrBNV_gp59-like protein	70656	70907	84	forward	76	98.8	79	98.8	75	98.8
76	GrBNV_gp60-like protein	71655	71068	196	reverse	77	99.49	80	99.49	76	100
77	hypothetical protein	71786	72115	110	forward	78	58.82	81	80.73		
78	polysaccharide lyase family 6-like protein	73738	72437	433	reverse	79	99.1	83/84	99.49/97.56	83/84	99.48/97.56
79	GrBNV_gp61-like protein	74565	73996	189	reverse	80	100	86	100	80	100
80	38K	74528	75367	279	forward	81	100	87	100	81	100
81	hypothetical protein	75371	75601	76	forward	82	100	88	100	82	100
82	hypothetical protein	76034	75747	96	reverse	83	97.9	89	98.95	83	98.95
83	GrBNV_gp28-like protein	79558	76382	1059	reverse	84	99.24	90	99.53	84	99.34
84	hypothetical protein	80265	80468	67	forward	85	64.81	92	100	85	100
85	hypothetical protein	80642	80445	65	reverse	86	98.46	93	100	86	100
86	hypothetical protein	80887	81201	105	forward	87	98.08	94	100	87	99.04
87	GrBNV_gp09-like protein	81265	82518	418	forward	88	99.76	95	99.76	88	99.76
88	LEF-9	84362	82692	556	reverse	89	99.8	96	99.82	89	99.64
89	mRNA decapping enzyme 2-like protein	84244	85029	262	forward	90	99.62	97	99.24	90	100
90	hypothetical protein	85613	85152	154	reverse	91	100	98	100	91	100
91	hypothetical protein	86103	86264	54	forward	94	100	100	100	93	100
92	hypothetical protein	86689	86408	94	reverse	95	100	101	100	94	98.925
93	RR2	86710	87912	401	forward	96	99.75	102	100	95	99.75
94	hypothetical protein	88095	89180	361	forward	97	99.72	103	98.61	96	99.72
95	GrBNV_gp62-like protein	89464	89177	96	reverse	98	100	104	98.95	97	100
96	GrBNV_gp43-like protein	90588	89521	356	reverse	99	99.72	105	99.72	98	99.72
97	VP91	90681	92660	660	forward	100	99.85	106	100	99	99.85
98	PIF-3	92703	93317	205	forward	101	100	107	100	100	100
99	DNA helicase 2	95848	93386	821	reverse	102	100	108	100	101	100
100	hypothetical protein	96427	96693	88		103	98.86	109	98.86	102	98.86
101	hypothetical protein	96716	96925	70	forward	104	100	110	100	103	100
102	hypothetical protein	97181	97333	50	forward	105	98	111	100	104	100
103	hypothetical protein	97484	97636	50	forward	106	96	112	98	105	98
104	P33	99015	97741	425	reverse	107	100	113	99.76	106	100
105	GrBNV_gp06-like protein	99181	100596	472	forward	108	99.79	114	99.79	107	99.58
106	PIF-5	100809	102044	412	forward	109	100	115	99.76	108	99.52
107	GrBNV_gp33-like protein	103102	102041	354	reverse	110	100	116	100	109	100
108	hypothetical protein	104171	103155	338	reverse	111	100	117	100	110	100
109	GrBNV_gp35-like protein	104170	104961	264	forward	112	100	118	100	111	100
110	GrBNV_gp36-like protein	105022	106152	376	forward	113	100	119	100	112	100
111	GrBNV_gp37-like protein	108260	106215	682	reverse	114	99.41	120	99.71	113	99.71
112	DNA ligase	109429	108356	358	reverse	115	100	121	100	114	100
113	GrBNV_gp39-like protein	109460	109942	161	forward	116	99.38	122	99.38	115	99.38
114	GrBNV_gp41-like protein	110375	109947	143	reverse	117	100	123	100	116	100
115	hypothetical protein	111600	110356	415	reverse	118	98.79	124	98.55	117	99.03
116	GrBNV_gp44-like protein	112682	111663	340	reverse	119	99.71	125	99.41	118	99.71
117	P74	112796	115006	737	forward	120	100	126	99.86	119	99.86
118	hypothetical protein	115358	115191	56	reverse	121	100	127	98.18	120	100
119	hypothetical protein	116292	116002	96	reverse			128	45.56	121	50
120	hypothetical protein	116511	118325	605	forward	123	91.26	134	91.69	122	90.89
121	hypothetical protein	118491	119333	281	forward	124	98.57	133	98.25	123	98.57
122	GrBNV_gp48-like protein	121176	119413	588	reverse	125	99.49	132	98.82	124	99.49
123	hypothetical protein	121228	121770	180	forward	126	98.32	131	95.6	125	95.6
124	hypothetical protein	121795	122004	70	forward	127	100	130	100		
125	hypothetical protein	122001	122231	76	forward	128	100				
126	hypothetical protein	123415	122312	368	reverse	129	98.91	136	98.37	127	99.73
127	GrBNV_gp17-like protein	123469	124932	487	forward	130	99.8	137	99.79	128	99.79
128	hypothetical protein	125079	125546	156	forward	131	100	138	100	129	100
129	hypothetical protein	125880	125641	79	reverse	132	100	139	100	130	100
