# Supplementary material for: Confirmation of Oryctes rhinoceros nudivirus infections in G-haplotype coconut rhinoceros beetles (Oryctes rhinoceros) from Palauan PCR-positive populations
Source: Sci Rep. 2021 Sep 20;11:18820. doi: 10.1038/s41598-021-97426-w (PMC8452681; doi:10.1038/s41598-021-97426-w)
Supplement: Supplementary file 3 — Supplementary Table S2. [file 41598_2021_97426_MOESM3_ESM.pdf]

Table S2. Open reading frames of isolates OrNV-X2B

OrNV-X2B ORF						Homologs in other isolates			
						OrNV-Ma07		OrNV-SI	
**ORF No.**	**Annotation**	**Start**	**End**	**aa**	**Direction**	ORF No.	%Identity	ORF No.	%Identity
1	dnapol B	1	3843	1281	forward	1	99.92	1	99.92
2	putative trypsin-like serine protease	3898	4917	340	forward	2	100	2	100
3	GrBNV_gp13-like protein	4928	6043	371	forward	3	99.73	3	99.73
4	Ac81-like protein	6067	6564	165	forward	4	99.39	4	99.39
5	Arenavirus nucleocapsid C-terminal domain-like protein	7155	6583	572	reverse	5	99.47	5	100
6	thymidylate synthase/pyrimidine hydroxymethylase-like protein	8167	7202	322	reverse	6	100	6	100
7	putative calcineurin-like phosphoesterase	9045	8212	332	reverse	7	100	7	100
8	hypothetical protein	9304	9137	55	reverse	8	100	8	100
9	NeseNPVORF52-like protein	9303	9968	222	forward	9	99.55	9	100
10	hypothetical protein	10197	9997	67	reverse	10	92.31	10	90.77
11	mitochondrial carrier protein-like protein	10334	11122	263	forward	11	100	11	99.62
12	odv-e66-like protein	12933	11152	604	reverse	12	99.66	12	100
13	hypothetical protein	13587	12985	201	reverse	13	100	13	100
14	hypothetical protein	13799	13590	69	reverse	14	100	14	100
15	vp39-like protein	14472	13720	251	reverse	15	100	15	100
16	polh/gran	14540	15886	449	forward	16	99.78	16	100
17	pif-2 protein	17043	15919	375	reverse	17	100	17	100
18	GrBNV_gp67-like protein	17184	18293	370	forward	18	99.46	18	100
19	Ac146-like protein	18315	18626	103	forward	19	99.03	19	100
20	p47 protein	18538	19512	324	forward	20	100	20	100
21	hypothetical protein	20607	19582	341	reverse	21	100	21	100
22	GrBNV_gp72-like protein	20606	21331	242	forward	22	99.18	22	100
23	guanylate kinase-like protein	22216	21458	253	reverse	23	100	23	100
24	GrBNV_gp75-like protein	22837	22256	193	reverse	24	100	24	100
25	GrBNV_gp76-like protein	22836	24494	553	forward	25	100	25	99.82
26	hypothetical protein	24555	25718	388	forward	26	100	26	100
27	GrBNV_gp78-like protein	26959	25754	402	reverse	27	100	27	100
28	hypothetical protein	26990	27826	279	forward	28	100	28	100
29	GrBNV_gp81-like protein	28617	27838	259	reverse	29	100	29	100
30	vlf-1	28484	30613	710	forward	30	99.3	30	99.72
31	hypothetical protein	31045	30875	57	reverse	31	100	31	100
32	19 kDa protein	32493	31726	256	reverse	33	99.61	32	99.61
33	dnahel	32608	36330	1241	forward	34	100	33	99.84
34	hypothetical protein	36703	36410	98	reverse	35	100	34	100
35	hypothetical protein	37141	36719	141	reverse	36	99.29	35	100
36	61k AcORF9	38128	37202	309	reverse	37	99.35	36	100
37	hypothetical protein	38441	38160	93	reverse	38	100	37	100
38	GrBNV_gp93-like protein	39095	38526	190	reverse	39	100	38	100
39	GrBNV_gp94-like protein	39069	39467	132	forward	40	100	39	100
40	GrBNV_gp95-like protein	39439	39780	114	forward	41	99.12	40	100
41	lef-4 protein	39756	40961	402	forward	42	99.75	41	100
42	hypothetical protein	41108	40953	51	reverse	43	100	42	100
43	GrBNV_gp97-like protein	41070	41660	197	forward	44	100	43	100
44	GrBNV_gp23-like protein	42644	41673	324	reverse	45	100	44	100
45	GrBNV_gp22-like protein	44268	42697	524	reverse	46	100	45	100
46	GrBNV_gp19-like protein	45385	44474	303	reverse	47	100	46	100
47	hypothetical protein	45893	45732	53	reverse	48	100	47	100
48	hypothetical protein	46204	45992	71	reverse	49	97.18	48	100
49	rr1 protein	46613	48826	738	forward	51	99.86	49	100
50	lef-5	49125	48889	78	reverse	52	100	50	100
51	GrBNV_gp84-like protein	49816	49169	215	reverse	53	100	51	100
52	GrBNV_gp83-like protein	50470	51789	439	forward	54	99.77	52	100
53	hypothetical protein	51970	51773	65	reverse	55	100	53	100
54	hypothetical protein	53492	52149	448	reverse	56	100	54	100
55	patatin-like phospholipase-like protein	53625	54734	370	forward	57	99.73	55	100
56	HZV_115-like protein	55402	54773	210	reverse	58	100	56	100
57	lef-3 protein	56005	55487	173	reverse	59	100	57	100
58	pif-1 protein	57584	56109	492	reverse	60	99.8	58	99.8
59	GrBNV_gp51-like protein	57669	58016	116	forward	61	97.39	59	97.39
60	hypothetical protein	58381	58115	89	reverse	62	100	60	100
61	hypothetical protein	58335	58556	73	forward	63	100	61	100
62	lef-8 protein	61350	58582	932	reverse	64	100	62	100
63	hypothetical protein	61818	61636	61	reverse	65	100	63	100
64	hypothetical protein	62325	62032	97	reverse	66	86.36		
65	hypothetical protein	62308	62559	84	forward	67	98.8	64	98.8
66	hypothetical protein	62870	62526	115	reverse	68	76.56		
67	FIC protein-like protein	63172	63834	221	forward	69	100	65	100
68	hypothetical protein	64152	64829	226	forward	71	99.56	67	100
69	Ac68-like protein	65421	65011	137	reverse	72	100	68	100
70	hypothetical protein	65697	66482	261	forward	73	99.23	69	100
71	densorvirus NS3-like protein	66610	67482	291	forward	74	98.97	70	100
72	integrase/recombinase-like protein	68623	67529	365	reverse	75	100	71	100
73	GrBNV_gp58-like protein	68670	68825	52	forward	76	100	72	100
74	semaphorin-like protein	68918	69286	123	forward	77	100	73	100
75	semaphorin-like protein	69205	70599	421	forward	78	100	74	100
76	GrBNV_gp59-like protein	70610	70861	84	forward	79	100	75	100
77	GrBNV_gp60-like protein	71596	71009	196	reverse	80	98.98	76	99.49
78	hypothetical protein	71727	72035	102	forward	81	85.29		
79	polysaccharide lyase family 6-like protein	73637	72336	433	reverse	83/84	99.49/98.78	77/78	99.49/98.78
80	GrBNV_gp61-like protein	74464	73895	189	reverse	86	100	80	100
81	38K protein	74427	75266	268	forward	87	100	81	100
82	hypothetical protein	75270	75500	77	forward	88	100	82	100
83	hypothetical protein	75933	75646	96	reverse	89	98.95	83	98.95
84	GrBNV_gp28-like protein	79434	76216	1074	reverse	90	98.06	84	99.16
85	hypothetical protein	80141	80491	116	forward	92	64.81	85	64.81
86	hypothetical protein	80612	80415	65	reverse	93	98.46	86	98.46
87	hypothetical protein	80857	81171	105	forward	94	98.08	87	99.04
88	GrBNV_gp09-like protein	81235	82488	418	forward	95	100	88	100
89	lef-9 protein	84350	82680	556	reverse	96	99.64	89	99.82
90	mRNA decapping protein 2-like protein	84232	85017	262	forward	97	98.86	90	99.62
91	hypothetical protein	85598	85137	154	reverse	98	100	91	100
92	Ac120-like protein	85795	85592	67	reverse	99	92.42	92	91.04
93	hypothetical protein	85812	86075	88	forward	131	85.71	125	85.71
94	hypothetical protein	86098	86259	54	forward	100	100	93	100
95	hypothetical protein	86684	86403	94	reverse	101	100	94	98.93
96	rr2 protein	86705	87907	401	forward	102	99.75	95	100
97	hypothetical protein	88092	89177	361	forward	103	98.89	96	100
98	GrBNV_gp62-like protein	89461	89174	96	reverse	104	98.95	97	100
99	GrBNV_gp43-like protein	90585	89518	356	reverse	105	100	98	100
100	vp91 protein	90678	92657	660	forward	106	99.85	99	100
101	pif-3 protein	92700	93314	205	forward	107	100	100	100
102	DNA helicase 2	95847	93385	821	reverse	108	100	101	100
103	hypothetical protein	96421	96687	88	forward	109	97.73	102	100
104	hypothetical protein	96710	96919	70	forward	110	100	103	100
105	hypothetical protein	97176	97328	50	forward	111	98	104	98
106	hypothetical protein	97499	97651	50	forward	112	98	105	98
107	Ac92-like protein	99030	97756	425	reverse	113	99.76	106	100
108	GrBNV_gp06-like protein	99196	100611	472	forward	114	100	107	99.79
109	PIF-5	100824	102059	412	forward	115	99.76	108	99.52
110	GrBNV_gp33-like protein	103117	102056	354	reverse	116	100	109	100
111	thymidylate kinase-like protein	104186	103170	338	reverse	117	100	110	100
112	GrBNV_gp35-like protein	104185	104976	264	forward	118	100	111	100
113	GrBNV_gp36-like protein	105037	106167	376	forward	119	100	112	100
114	GrBNV_gp37-like protein	108275	106230	682	reverse	120	99.71	113	99.71
115	DNA ligase	109446	108373	358	reverse	121	100	114	100
116	GrBNV_gp39-like protein	109477	109959	161	forward	122	100	115	100
117	GrBNV_gp41-like protein	110392	109964	143	reverse	123	100	116	100
118	hypothetical protein	111617	110373	415	reverse	124	99.04	117	99.76
119	GrBNV_gp44-like protein	112699	111680	340	reverse	125	99.71	118	100
120	p74 protein	112813	115023	737	forward	126	99.86	119	99.86
121	hypothetical protein	115375	115208	56	reverse	127	98.18	120	100
122	hypothetical protein	115589	115398	63	reverse	109	82.35	102	82.35
123	iap-3	116441	118216	592	forward	134	99.46	122	98.91
124	hypothetical protein	118382	119212	277	forward	133	96.84	123	100
125	GrBNV_gp48-like protein	121055	119292	588	reverse	132	98.32	124	99.66
126	hypothetical protein	121107	121484	125	forward	131	94.96	125	100
127	hypothetical protein	121675	121884	69	forward	130	100	126	100
128	hypothetical protein	121881	122111	76	forward				
129	hypothetical protein	123286	122192	365	reverse	136	98.9	127	99.18
130	GrBNV_gp17-like protein	123340	124803	487	forward	137	100	128	100
131	hypothetical protein	124950	125417	156	forward	138	100	129	100
132	hypothetical protein	125746	125507	79	reverse	139	100	130	100
